# Ubiquitin-specific protease 14 modulates degradation of cellular prion protein

**DOI:** 10.1038/srep11028

**Published:** 2015-06-10

**Authors:** Takujiro Homma, Daisuke Ishibashi, Takehiro Nakagaki, Takayuki Fuse, Tsuyoshi Mori, Katsuya Satoh, Ryuichiro Atarashi, Noriyuki Nishida

**Affiliations:** 1 Department of Molecular Microbiology and Immunology, Nagasaki University Graduate School of Biomedical Sciences, Nagasaki, Japan

## Abstract

Prion diseases are fatal neurodegenerative disorders characterized by the accumulation of prion protein (PrP^C^). To date, there is no effective treatment for the disease. The accumulated PrP, termed PrP^Sc^, forms amyloid fibrils and could be infectious. It has been suggested that PrP^Sc^ is abnormally folded and resistant to proteolytic degradation, and also inhibits proteasomal functions in infected cells, thereby inducing neuronal death. Recent work indicates that the ubiquitin-proteasome system is involved in quality control of PrP^C^. To reveal the significance of prion protein ubiqitination, we focused on ubiquitin-specific protease 14 (USP14), a deubiqutinating enzyme that catalyzes trimming of polyubiquitin chains and plays a role in regulation of proteasomal processes. Results from the present study showed that treatment with a selective inhibitor of USP14 reduced PrP^C^, as well as PrP^Sc^, levels in prion-infected neuronal cells. Overexpression of the dominant negative mutant form of USP14 reduced PrP^Sc^, whereas wildtype USP14 increased PrP^Sc^ in prion-infected cells. These results suggest that USP14 prevents degradation of both normal and abnormal PrP. Collectively, a better understanding about the regulation of PrP^Sc^ clearance caused by USP14 might contribute greatly to the development of therapeutic strategies for prion diseases.

Prion diseases are fatal neurodegenerative disorders associated with the conformational conversion of cellular prion protein (PrP^C^) to β-sheet-rich abnormal prion protein (PrP^Sc^)^1^. PrP^C^ builds up in the rough endoplasmic reticulum (ER), is transported to the Golgi, and subsequently sorted to the cell surface as a glycosylphosphatidylinositol (GPI)-anchored protein. During its biosynthetic maturation in the ER, PrP^C^ is subject to several posttranslational modifications, including cleavage of the N-terminal signal peptide and C-terminal hydrophobic peptide, addition of N-linked oligosaccharide chains at two sites, formation of a single disulphide bond, and attachment of the GPI anchor^2^,^3^,^4^.

Misfolded proteins are removed from the ER by retrograde transportation to the cytosol and degraded by the ubiquitin–proteasome system, which is referred to as endoplasmic reticulum-associated degradation (ERAD). A recent study revealed that ubiquitin ligase gp78 is involved in unglycosylated prion protein degradation^5^. However, to the best of our knowledge, there is as yet no convincing evidence demonstrating direct ubiquitination of prion protein. Although both nascent wildtype^6^,^7^ and misfolded PrP^8^,^9^,^10^ are actually degraded by ERAD prior to release into the secretory pathway, the precise mechanism by which proteasome degrades prion protein has not been established.

Ubiquitin-specific protease 14 (USP14), one of the deubiquitinating enzymes, is localized at the proteasome^11^. USP14 plays a special role in rescuing proteins from degradation by trimming ubiquitin chains from its substrate-distal tip of the ubiquitinated protein^12^,^13^,^14^. In yeast and mammals, loss of USP14/Ubp6 (yeast homolog of USP14) results in increased degradation of ubiquitinated proteins and reduced levels of free ubiquitin, suggesting that USP14 is required for ubiquitin recycling at the proteasome to maintain ubiquitin pools^15^,^16^,^17^,^18^. Thus, the proteasomal function is limited by USP14-dependent chain trimming. It has been shown that endogenous USP14 negatively regulates ERAD^19^. USP14 overexpression inhibits degradation of the null Hong Kong mutant α1-antitrypsin, an ER luminal unfolded protein of ERAD substrate^20^, whereas USP14 depletion by small interfering RNA effectively accelerates its degradation. Intriguingly, Lee *et al.* demonstrated that IU1, a selective small-molecule inhibitor of USP14, accelerated proteasomal degradation of tau and TDP-43, which have been implicated in neurodegenerative diseases^21^. Thus, we focused on the USP14 system to reveal PrP^C^ degradation in the proteasome, and investigated whether USP14 is related to regulation of prion protein degradation. Results demonstrated that an inhibitor of USP14 reduced PrP^C^ in mouse neuroblastoma cells, as well as PrP^Sc^, indicating that USP14 negatively regulates degradation of prion protein.

## Results

### Effects of IU1 treatment on prion protein degradation

**Figure 1:**
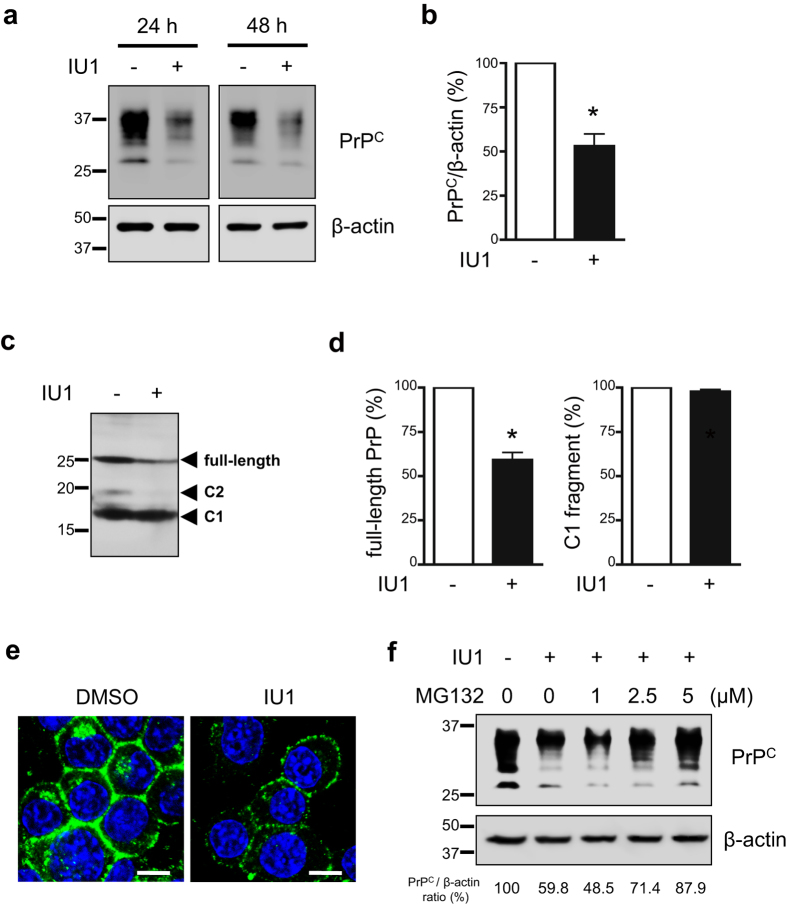
IU1 treatment reduces PrP^C^ levels in N2a58 cells. (**a**) N2a58 cells were treated with DMSO or 100 μM IU1 for 24 or 48 h. Lysates were analyzed by immunoblotting with anti-PrP (SAF32) and anti-β-actin antibodies. (**b**) The graph shows the PrP^C^ levels in N2a58 cells after treatment with DMSO or 100 μM IU1 for 48 h from at least three independent experiments. Asterisk indicates significant difference (*P < 0.05). Mean ± SD. (**c**) N2a58 cell lysates were deglycosylated with PNGase F followed by immunoblotting with anti-PrP (SAF83) antibody. (**d**) Quantification of deglycosylated PrP from at least three independent experiments was performed as described in (**c**). Asterisk indicates significant difference (*P < 0.05). Mean ± SD. (**e**) N2a58 cells were treated with DMSO or 100 μM IU1 for 48 h. PrP^C^ (SAF32; green) and nuclei (blue) were visualized. Bars: 10 μm. (**f**) During 48 h of IU1 treatment and 12 h prior to harvest, N2a58 cells were treated with DMSO or MG132 (1, 2.5, 5 μM). Lysates were analyzed by immunoblotting with anti-PrP (SAF32) and anti-β-actin antibodies. Numbers below the gel indicate relative expression of PrP^C^ normalized to β-actin.

To examine the effect of IU1 on PrP^C^ degradation, mouse neuroblastoma N2a cells expressing endogenous PrP (N2a58 cells) were treated with 100 μM IU1, an agent that inhibits the function of USP14^21^, for 24 or 48 h. Upon lysis, we observed that protein levels of PrP^C^ were reduced in IU1-treated N2a58 cells compared with DMSO-treated N2a58 cells (Fig. 1a,b). The mRNA levels of PrP^C^ were not altered after the treatment for 48 h (Supplementary Fig. S1a). Reduction of protein levels of PrP^C^ was also observed in mouse hypothalamic GT1-7 cells (Supplementary Fig. S2), indicating that the effect was not restricted to N2a58 cells.

For better signal comparison, proteins were deglycosylated with PNGase F. After deglycosylation, we observed full-length PrP, the highest level of C1 fragments, and barely detectable levels of C2 fragments (Fig. 1c). Of note, IU1 treatment significantly reduced full-length PrP levels, but had little effect on C1 fragment levels (Fig. 1c,d). We next analyzed PrP^C^ in cells using immunofluorescence. N2a58 cells were treated with 100 μM IU1 for 48 h and stained with anti-PrP antibody for PrP^C^ detection. As expected, we observed reduced PrP^C^ levels after IU1 treatment (Fig. 1e). To confirm whether PrP^C^ degradation was proteasome-dependent, we utilized the proteasome inhibitor MG132. During 48 h of IU1 treatment and 12 h prior to harvest, vehicle DMSO or MG132 was added to the cultures. As expected, in the presence of MG132, PrP^C^ degradation was prevented in a dose-dependent manner (Fig. 1f).

Autophagy is one of the major cellular degradation pathways involved in protein and organelle turnover, in addition to proteasomal degradation. Therefore, we examined the effect of IU1 on autophagic flux by measuring protein levels of an autophagosomal membrane protein LC3-II, which is involved in autophagosome formation. IU1 treatment had little effect on protein levels of LC3-II, while the lysosome inhibitor (NH_4_Cl) treatment increased LC3-II levels in N2a58 cells (Supplementary Fig. S3). Furthermore, during 48 h of IU1 treatment and 24 h prior to harvest, NH_4_Cl was added to the cultures. The combination of IU1/NH_4_Cl resulted in no further increase of LC3-II, suggesting that PrP^C^ reduction by IU1 treatment was not due to autophagy activation. Taken together, these results demonstrate that IU1 treatment reduces PrP^C^ via proteasome-dependent degradation.

**Figure 2:**
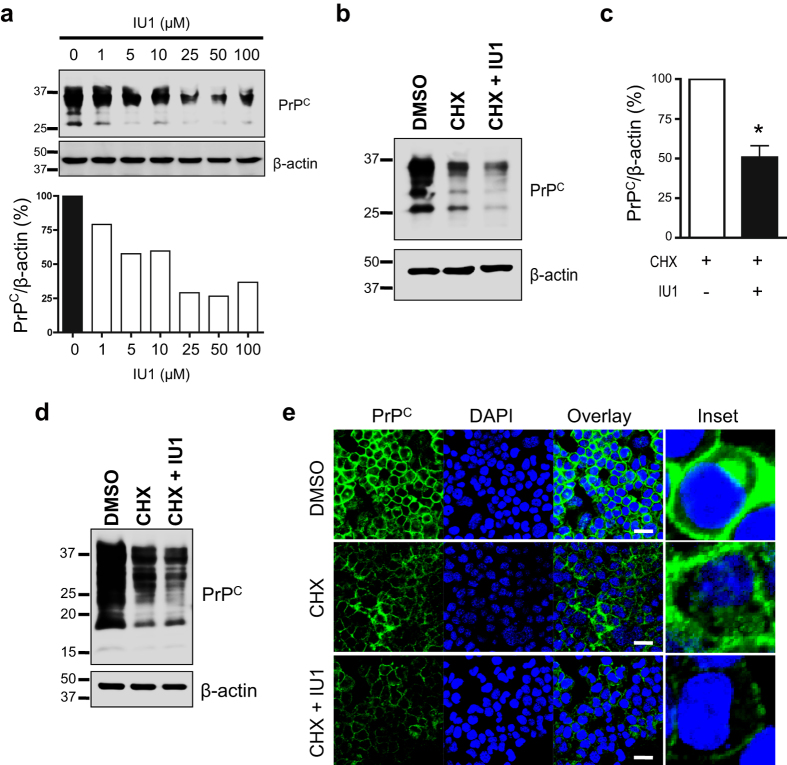
IU1 treatment accelerates degradation of PrP^C^ in N2a58 cells. (**a**) N2a58 cells were treated with 50 μg/ml CHX combined with various concentrations of IU1 (1–100 μM) for 6 h. Lysates were analyzed by immunoblotting with anti-PrP (SAF32) and anti-β-actin antibodies. (**b**) N2a58 cells were treated with DMSO, 50 μg/ml CHX alone, or a combination of CHX and 100 μM IU1 for 6 h. Lysates were analyzed by immunoblotting with anti-PrP (SAF32) and anti-β-actin antibodies. (**c**) Quantification of PrP^C^ from at least three independent experiments was performed as described in (**b**). Asterisk indicates significant difference (*P < 0.05). Mean ± SD. (**d**) HpL cells were transfected with pcDNA3.1 mouse PrP expression plasmid. After 48 h, cells were treated with DMSO, 50 μg/ml CHX alone, or a combination of CHX and 100 μM IU1 for 6 h. Lysates were analyzed by immunoblotting with anti-PrP (SAF83) and anti-β-actin antibodies. (**e**) N2a58 cells were treated with DMSO, 50 μg/ml CHX alone, or a combination of CHX and 100 μM IU1 for 6 h. PrP^C^ (SAF32; green) and nuclei (blue) were visualized. Bars: 20 μm.

To analyze PrP^C^ protein stability, N2a58 cells were treated with the protein synthesis inhibitor cycloheximide (CHX) combined with various concentrations of IU1 (1–100 μM) for 6 h and subsequently analyzed by immunoblotting with anti-PrP antibody. The reduction of PrP^C^ levels by IU1 treatment was dose-dependent and detectable at IU1 concentrations as low as 1 μM (Fig. 2a). To test whether IU1 treatment could accelerate proteasomal degradation of PrP^C^, N2a58 cells were treated with CHX alone or in combination with 100 μM IU1 for 6 h, and the lysates were subsequently subjected to immunoblotting with anti-PrP antibody. CHX treatment reduced PrP^C^ level in N2a58 cells (Fig. 2b). Of note, CHX/IU1 combination reduced PrP^C^ more significantly than CHX treatment alone (Fig. 2b,c). Similar results were obtained in PrP-deficient HpL mouse hippocampal cells transiently transfected with PrP, according to immunoblot results (Fig. 2d). Immunofluorescence analysis revealed that CHX/IU1 combination reduced PrP^C^ level more significantly than CHX treatment alone in N2a58 cells (Fig. 2e). These findings indicated that proteasomal degradation of PrP^C^ was indeed dramatically stimulated by IU1 treatment.

**Figure 3:**
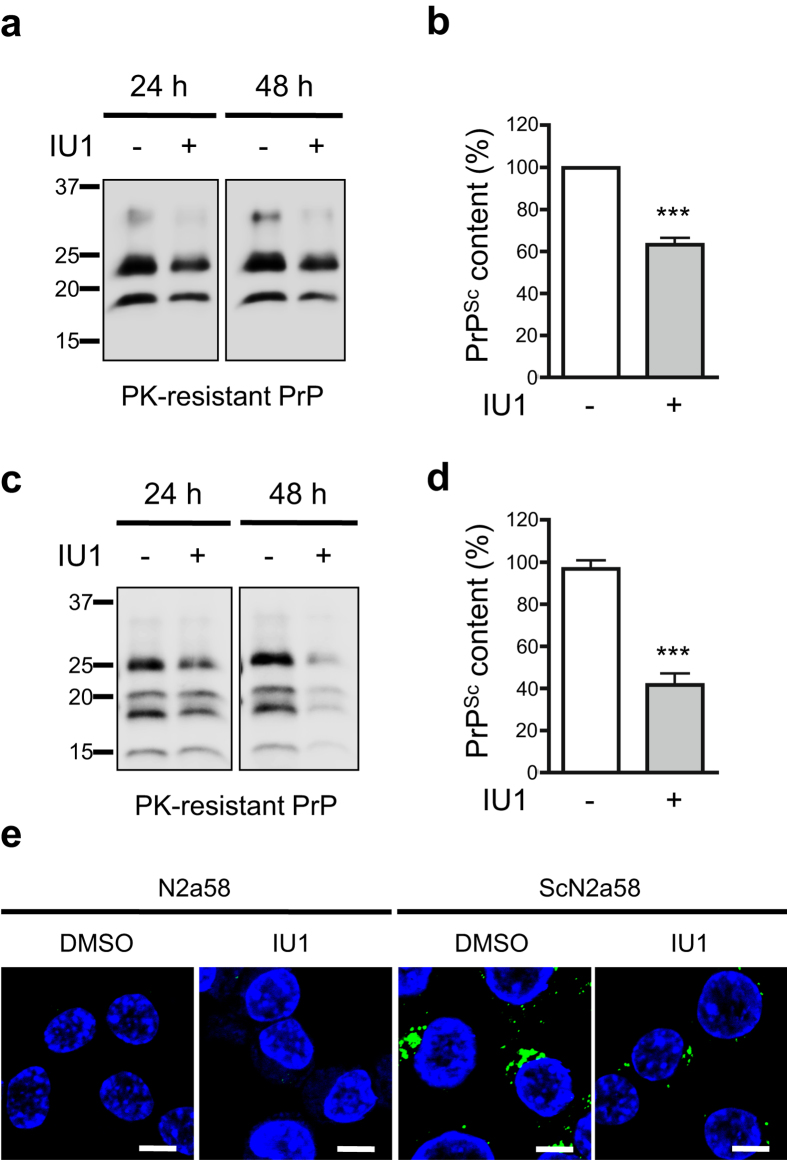
IU1 treatment reduces PrP^Sc^ levels in prion-infected cells. (**a**) ScN2a58 cells were treated with DMSO or 100 μM IU1 for 24 or 48 h. Lysates were PK-digested followed by immunoblotting with anti-PrP (M20) antibody. (**b**) Quantification of PrP^Sc^ after IU1 treatment for 48 h from at least three independent experiments performed as described in (**a**). Asterisk indicates significant difference (***P < 0.0001). Mean ± SD. (**c**) N2a58-FK cells were treated with DMSO or 100 μM IU1 for 24 or 48 h. Lysates were PK-digested followed by immunoblotting with anti-PrP (M20) antibody. (**d**) Quantification of PrP^Sc^ after IU1 treatment for 48 h from at least three independent experiments performed as described in (**c**). Asterisk indicates significant difference (***P < 0.0001). Mean ± SD. (**e**) N2a58 or ScN2a58 cells were treated with DMSO or 100 μM IU1 for 48 h. PrP^Sc^ (mAb132; green) and nuclei (blue) were visualized. Bars: 10 μm.

For further experiments, we utilized ScN2a58 cells or FK-N2a58 cells, and persistently scrapie-derived 22 L or Gerstmann-Sträussler-Scheinker (GSS) syndrome-derived Fukuoka-1 strain infected N2a58 cells, respectively. Immunoblotting revealed that IU1 treatment reduced PrP^Sc^ levels in ScN2a58 cells (Fig. 3a,b). IU1 treatment also reduced PrP^Sc^ levels in FK-N2a58 cells (Fig. 3c,d). To support this, we visualized PrP^Sc^ using mAb132, which is a specific monoclonal antibody that reacts with PrP^Sc^ in immunofluorescence analysis^22^. We confirmed reduced PrP^Sc^ levels in ScN2a58 cells after IU1 treatment (Fig. 3e). The mRNA levels of PrP^C^ in ScN2a58 cells were not altered after IU1 treatment for 48 h (Supplementary Fig. S1b). Taken together, these results demonstrate that IU1 treatment reduced PrP^Sc^ levels in prion-infected cells.

### USP14 negatively regulates prion protein degradation

**Figure 4:**
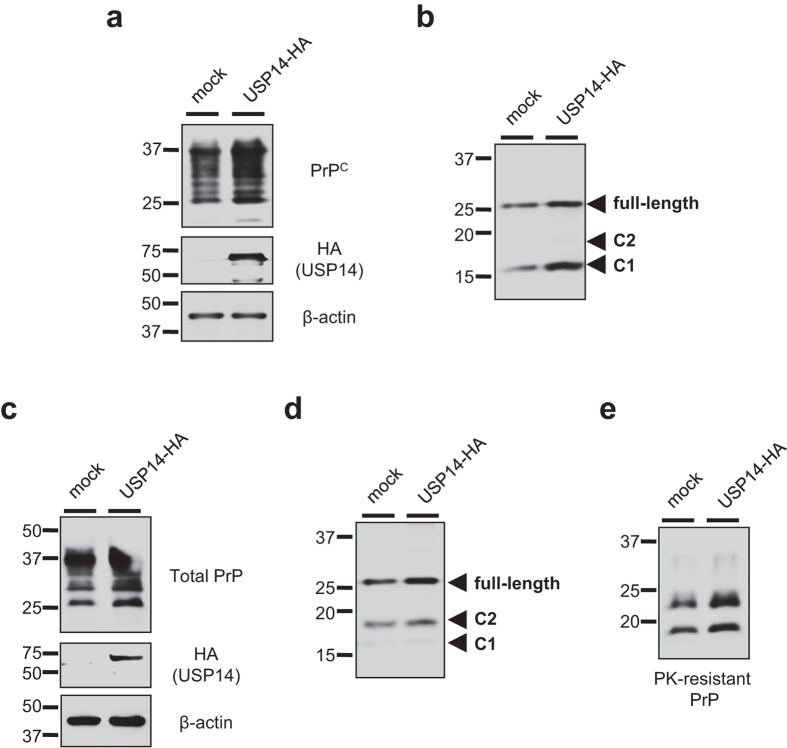
Overexpression of USP14 increases PrP^Sc^ levels in prion-infected cells. (**a**) N2a58 cells were transfected with an empty plasmid (mock) or a HA-tagged USP14 expression plasmid for 48 h. Lysates were analyzed by immunoblotting with anti-PrP (SAF32), anti-HA, and anti-β-actin antibodies. (**b**) Mock or USP14-overexpressed N2a58 cell lysates were deglycosylated with PNGase F followed by immunoblotting with anti-PrP (SAF83) antibody. (**c**) Mock or USP14-overexpressed ScN2a58 cells were analyzed by immunoblotting with anti-PrP (SAF32), anti-HA, and anti-β-actin antibodies. (**d**) Mock or USP14-overexpressed ScN2a58 cell lysates were deglycosylated with PNGase F followed by immunoblotting with anti-PrP (SAF83) antibody. (**e**) Mock or USP14-overexpressed ScN2a58 cell lysates were PK-digested followed by immunoblotting with anti-PrP (M20) antibody.

To investigate whether USP14 overexpression could influence prion protein degradation, N2a58 and ScN2a58 cells were transiently transfected with a HA-tagged USP14 expression plasmid for 48 h and subjected to immunoblotting. As expected, we confirmed that total PrP^C^ levels (Fig. 4a), deglycosylated full-length PrP (Fig. 4b), and C1 fragment (Fig. 4b) levels increased in USP14-overexpressed cells compared to mock transfected N2a58 cells. The mRNA levels of PrP^C^ were not altered in USP14-overexpressed cells compared to mock transfected N2a58 cells (Supplementary Fig. S1c). Of note, we observed that total PrP (Fig. 4c), deglycosylated full-length PrP, and C2 fragment (Fig. 4d), as well as PrP^Sc^ (Fig. 4e), levels increased in USP14-transfected ScN2a58 cells.

**Figure 5:**
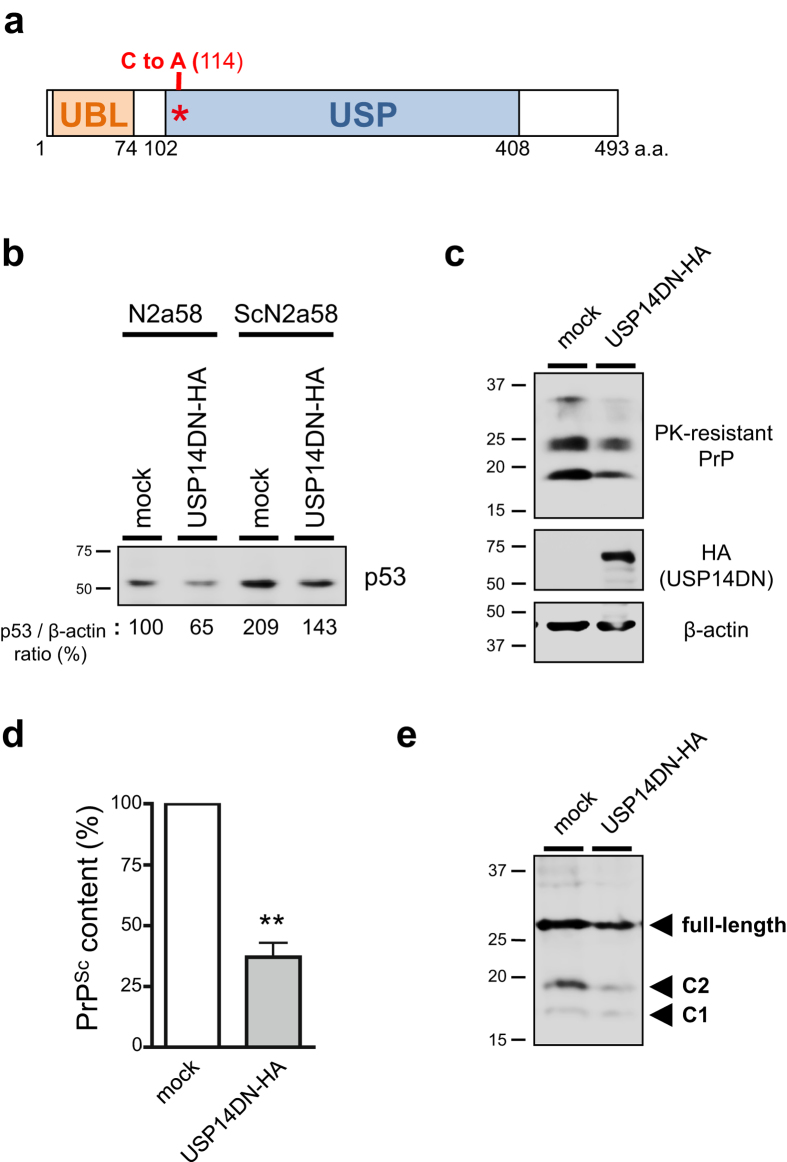
Overexpression of dominant negative USP14 reduces PrP^Sc^ levels in prion-infected cells. (**a**) Schematic representation of the dominant negative USP14 (USP14DN). USP14 contains the ubiquitin-like (UBL) domain, which is important for localization at the proteasome, as well as the ubiquitin-specific protease (USP) domain. Asterisk indicates substitution of mouse USP14 cysteine 114 for alanine in the USP domain. (**b**) N2a58 or ScN2a58 cells were transfected with an empty plasmid (mock) or a HA-tagged dominant negative USP14 (USP14DN) expression plasmid for 48 h. Lysates were analyzed by immunoblotting with anti-p53 and anti-β-actin antibodies. Numbers below the gel indicate relative expression of p53 normalized to β-actin. (**c**) Mock or USP14DN-overexpressed ScN2a58 cell lysates were analyzed by immunoblotting with anti-HA and anti-β-actin antibodies. Alternatively, lysates were PK-digested followed by immunoblotting with anti-PrP (M20) antibody. (**d**) Quantification of PrP^Sc^ from at least three independent experiments performed as in (**c**). Asterisk indicates significant difference (**P < 0.01). Mean ± SD. (**e**) Mock or USP14DN-overexpressed ScN2a58 cell lysates were deglycosylated with PNGase F followed by immunoblotting with anti-PrP (SAF83) antibody.

Conversely, to examine whether dominant negative USP14 could counteract PrP^Sc^ accumulation in prion-infected cells, a HA-tagged dominant negative USP14 (USP14DN), which was mutated by the substitution of cysteine with alanine at position 114 of USP region in mouse USP14 (Fig. 5a), was prepared. As a control study, we verified an effect of USP14DN against expression of p53 protein, a well-characterized proteasomal substrate, in cells. As expected, p53 proteins were also reduced in N2a58 and ScN2a58 cells transducing the USP14DN compared to mock groups (Fig. 5b). The mRNA levels of PrP^C^ were not altered in USP14DN-overexpressed cells compared to mock transfected N2a58 cells (Supplementary Fig. S1c). Of note, we detected significantly reduced PrP^Sc^ in the dominant negative-transfected cells compared to the mock-transfected control cells (Fig. 5c,d). We also confirmed that deglycosylated full-length PrP and C2 fragment levels were reduced in the dominant negative-transfected cells (Fig. 5e).

Finally, we analyzed endogenous USP14 protein expression by immunoblotting. No differences in USP14 expression were detectable between N2a58 and ScN2a58 cells (Supplementary Fig. S4). It is plausible that prion infection did not influence USP14 expression, because no variations in proteasome content or in its subunit composition were detected between scrapie-positive and control samples^23^, although prion infection inhibits the function^24^,^25^.

## Discussion

Previous studies have revealed that a series of prion proteins is degraded by ERAD (Step 1 in Supplementary Fig. S5). Wildtype PrP^C^ undergoes ERAD and is degraded by the proteasome^6^,^7^. Additionally, GSS-associated PrP mutant (Y145stop, Q217R and Q212P) and Creutzfeldt-Jakob disease-associated PrP mutant (V203, E211Q) are also subject to ERAD and degraded by the proteasome^8^,^9^,^10^. Overexpression of EDEM-3, a protein that recognizes N-linked glycans on aberrantly folded proteins and sorts them for ERAD, resulted in reduced PrP^Sc^ accumulation^26^. Alternatively, the proteasome also cotranslationally degraded nascent peptide chains. Other studies, however, have indicated that PrP is not subject to retrotranslocation from the ER into the cytoplasm prior to degradation by the proteasome^27^,^28^. During PrP biosynthesis, some PrP molecules fail to translocate into the ER lumen and are degraded by the proteasome due to the intrinsic inefficiency of signal peptides^27^,^28^.

Based on our results, we propose a model that shows that USP14 rescues prion protein from proteasomal degradation. When it comes to degradation, a certain population of prion proteins might escape from proteasomal degradation due to the presence of USP14 (Step 2). Our results demonstrated that USP14 overexpression increased total PrP levels (Fig. 4). In addition, we provide data that IU1 treatment accelerated prion protein degradation (Fig. 2), implicating the existence of the surplus prion protein. PrP cleavage is thought to occur either in endocytic vesicles^29^ or in the late secretory pathway^30^,^31^. In the current study, IU1 treatment reduced full-length PrP levels, but not C1 fragment levels (Fig. 1c,d), indicating that the affected PrP molecules appear to be within an earlier compartment of the secretory pathway. Although it is not abundant, the C2 fragment is apparently present in normal human brains^32^,^33^. It has been reported that C2 cleavage can be stimulated by reactive oxygen species^34^. We also barely detected C2 fragment, and it seems to be affected by IU1 treatment through unknown mechanisms (Fig. 1c).

PrP^Sc^ has been shown to be difficult to unfold and inhibits the proteasome^24^,^25^. Under circumstances in which the proteasome is inhibited, it facilitates conversion of cytosolic PrP^C^ to an abnormal, PrP^Sc^-like form, with partial protease resistance and detergent insolubility^35^. Hence, it is likely that IU1 treatment reduced PrP^C^ levels (Fig. 1), and this subsequently reduced further conversion of PrP^C^ to PrP^Sc^ in prion-infected cells (Fig. 3) (Step 3).

In summary, our findings demonstrated a novel mechanism by which USP14 negatively regulated prion protein degradation via the proteasome, although the detailed mechanism remains unclear. It has a possiblility that USP14 may regulate PrP degradation via an indirect mechanism because it remains controversial whether PrP is ubiquitinated and USP14 can affect its ubiquitination level. At the current moment, we speculate that it might be important for prion protein degradation that some ubiquitinated cargo proteins bind to PrP, thereby transporting PrP to the proteasome. However, further investigations are required for the elucidation of its opinion.

IU1 has been shown to accelerate proteasomal degradation of aggregation-prone proteins, including proteins associated with neurodegenerative diseases, such as tau, TDP-43, polyglutamine-expanded ataxin-3^21^, and abnormal prion protein in this study. These results suggest USP14 inhibition as a new therapeutic approach for the treatment of protein aggregation diseases through proteasomal degradation of disease-associated proteins.

## Materials and methods

### Antibodies

Anti-USP14 (Cell Signaling Technology, #11931), anti-β-actin (MBL, 177-3), anti-HA-tag (MBL, 561), anti-LC3B (Cell Signaling Technology, #2775), anti-p53 (Cell Signaling Technology, #2524), and anti-PrP (Santa Cruz Biotechnology, M20; SPI-Bio, SAF32 and SAF83) antibodies were purchased from the indicated vendors. The anti-PrP mAb132 was a kind gift from Prof. Motohiro Horiuchi (Hokkaido University). Horseradish peroxidase-conjugated anti-goat (Jackson ImmunoResearch), anti-mouse, and anti-rabbit (GE Healthcare Life Sciences) IgG antibodies were used for immunoblotting.

### Cell cultures

The mouse neuroblastoma Neuro 2a cells were obtained from the American Type Culture Collection (CCL 131). N2a58 cells are mouse PrP^C^-overexpressing Neuro 2a cells. ScN2a58 cells originated from N2a58 cells infected with a mouse-adapted scrapie strain, 22 L, as previously described^36^,^37^,^38^. FK-N2a58 cells originated from N2a58 cells infected with a mouse-adapted Gerstmann-Sträussler-Scheinker (GSS) strain, Fukuoka-1, as previously described^39^. HpL2-3 cells are immortalized hippocampal cells derived from PrP-deficient mice, a kind gift from Prof. Takashi Onodera (The University of Tokyo, Japan). The above cells were grown at 37 °C in 5% CO_2_ in Dulbecco’s-modified Eagle’s medium (DMEM; Sigma-Aldrich) containing 10% heat-inactivated fetal bovine serum (FBS), 100 units/ml penicillin, and 100 μg/ml streptomycin (Invitrogen). Immortalized mouse hypothalamic GT1-7 cells were maintained in DMEM containing 5% heat-inactivated FBS, 5% heat-inactivated horse serum, 100 units/ml penicillin, and 100 μg/ml streptomycin, as previously described^36^.

### Plasmids

The mouse PrP expression plasmid has been previously described^40^. The mouse USP14 open reading frame was amplified from N2a58 cDNA with primers: 5′-ATT GGG CCC CTC GCC ATG CCA CTC TAC TCT GTT ACA-3′ and 5′-CCG CTC GAG TCA TTA AGC GTA ATC TGG AAC ATC GTA TGG GTA CTG TTC ACT TTC TTC TTC-3′. To generate a dominant-negative mutant of USP14, the substitution of mouse USP14 cysteine 114 for alanine in the UBL domain was produced by PCR with primers: 5′-CTT GGT AAC ACT GCT TAC ATG AAT-3′ and 5′-ATT CAT GTA AGC AGT GTT ACC AAG-3′. Amplified PCR fragments were inserted into the ApaI and XhoI sites of expression plasmid pcDNA3.1 (Invitrogen) and confirmed by sequential analysis.

### Immunoblotting

Immunoblotting was performed as previously described^41^. For PrP^Sc^ detection, lysates were digested with 40 μg/ml of proteinase K (PK; Sigma-Aldrich) at 37 °C for 30 min. Bands were visualized using the ECL Western Blotting Detection Kit (GE Healthcare Life Sciences). Band intensities were quantified using ImageJ software (National Institutes of Health).

### Plasmid transfection

Transfection with plasmids was performed with PEI “Max” (Polysciences, Warrington). Four micrograms of PEI was used per microgram of transfected DNA. PEI and DNA were each added to 250 μl serum-free Opti-MEM, and the two solutions were combined and mixed by vortex. After a 15-min incubation at room temperature, the mixture was added to 2 ml DMEM in the well. After 48 h, lysates were prepared and subjected to immunoblotting.

### IU1 treatment

IU1, a selective inhibitor of USP14, was purchased from Focus Biomolecules. Cells were treated with dimethyl sulfoxide (DMSO) or 100 μM IU1 for 48 h and cell lysates were prepared, unless stated otherwise. Alternatively, cells were treated with 50 μg/ml cycloheximide (CHX; Sigma-Aldrich), combined with 100 μM IU1 for 6 h, and subjected to immunoblotting.

### Deglycosylation of proteins

Deglycosylation of proteins was performed according to manufacturer’s protocol (New England Biolabs). Briefly, proteins were denatured by boiling for 10 min in 0.5% SDS and 1% 2-mercaptoethanol. A 1/10-volume of 10% Nonidet P-40, 1/10-volume of 0.5 M sodium phosphate (pH 7.5), and 2 μl of PNGase F (500 units/μl) were added to each sample. After incubation for 2 h at 37 °C, the samples were subjected to immunoblotting.

### Real-time PCR

Total RNA was isolated from cells using a TRIZOL^®^ Reagent (Invitrogen) or GenElute™ Mammalian Total RNA Kit^®^ (Sigma). After the extracted total RNA was purified by RNeasy MinElute Cleanup Kit (QIAGEN) with RNase-free DNase set (QIAGEN), the first-strand cDNA was synthesized from 5 μg of total RNA with ThermoScript Reverse Transcriptase^®^ (Invitrogen). The primers were: 5′-GAT CCA TTT TGG CAA CGA CT-3′ and 5′-GTG TGC TGC TTG ATG GTG AT-3′ for mouse *prnp* gene and 5′-AAA TCG TGC GTG ACA TCA AA-3′ and 5′-AAG GAA GGC TGG AAA AGA GC-3′ for mouse *β-actin* gene. The level of β-actin mRNA was determined as a positive control and used to normalize as the mRNA expression level as an internal control. For real-time PCR, the synthesized cDNA was reacted with LightCycler^®^ 480 SYBR Green I Master (Roche Applied Science) and measured by Light Cycler 480 instrument (Roche Applied Science).

### Immunofluorescence analysis

Immunofluorescence staining was performed as previously described^41^. Cells were treated with DMSO or 100 μM IU1 for 48 h and washed twice in PBS followed by fixation using 4% formaldehyde for 30 min at room temperature. The cells were permeabilized in 0.5% Triton X-100 for 5 min. The slides were then incubated in blocking solution (5% nonfat dry milk) for 30 min at 37 °C. Incubation with primary antibodies (SAF32 for PrP^C^; mAb132 for PrP^Sc^) was performed overnight at 4 °C^22^. For PrP^Sc^ detection, the slides were treated with 3 M guanidine thiocyanate for 5 min at room temperature before staining. Alexa Fluor^®^ 488-conjugated anti–mouse IgG antibody (Invitrogen) served as the secondary antibody. Nuclei were stained with DAPI. All images were obtained using a confocal laser-scanning microscope LSM 700 (Carl Zeiss).

### Statistical analysis

Results in the graph represent mean ± standard deviation (SD) of at least three independent experiments. Statistical analysis was performed using GraphPad Prism 4 software. P-values were calculated using the Student’s *t*-test.

## Additional Information

**How to cite this article**: Homma, T. *et al.* Ubiquitin-specific protease 14 modulates degradation of cellular prion protein. *Sci. Rep.* **5**, 11028; doi: 10.1038/srep11028 (2015).

## Supplementary Material

Supplementary Information
