# Supplementary material for: Ubiquitin-specific protease 14 modulates degradation of cellular prion protein
Source: Sci Rep. 2015 Jun 10;5:11028. doi: 10.1038/srep11028 (PMC4462021; doi:10.1038/srep11028)
Supplement: Supplementary Information [file srep11028-s1.pdf]

Supplementary Information

Ubiquitin-specific protease 14 modulates degradation of cellular prion protein

Takujiro Homma, Daisuke Ishibashi, Takehiro Nakagaki, Takayuki Fuse, Tsuyoshi Mori, Katsuya Satoh, Ryuichiro Atarashi, and Noriyuki Nishida

Department of Molecular Microbiology and Immunology, Nagasaki University Graduate School of Biomedical Sciences, 1-12-4 Sakamoto, Nagasaki 852-8523, Japan

Supplementary Figure S1

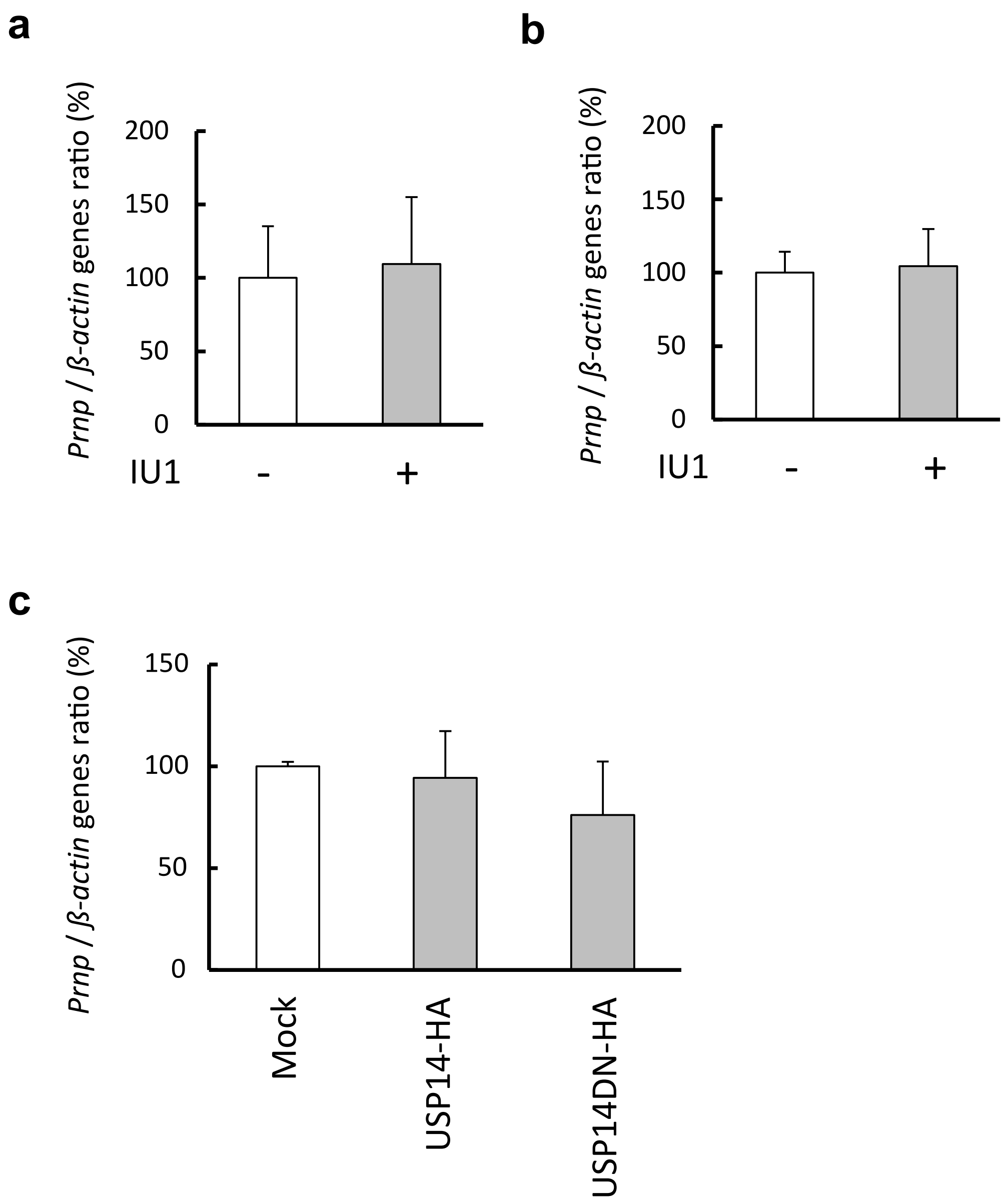

The level of *prnp* gene expression after IU1 treatment or USP14 transduction in N2a58 cells.
The level of *prnp* mRNA in N2a58 cells were analyzed by Real time PCR. N2a58 (a) and ScN2a58 (b) cells were treated with DMSO or 100 μM IU1 for 48 h. (c) N2a58 cells were transfected with an empty vector (Mock) or a HA-tagged USP14 or an USP14DN expression plasmid for 48 h. For mRNA quantification, the *β-actin* mRNA that is constitutively expressed, a so-called house-keeping gene, was used to normalize the mRNA expression level as an internal control.

Supplementary Figure S2

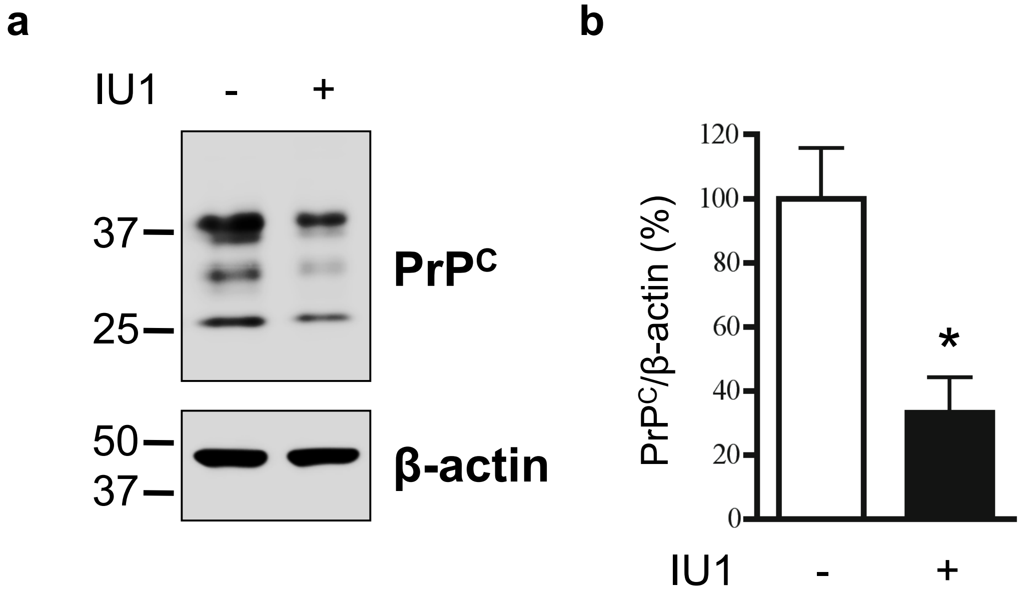

IU1 treatment reduces PrP^{C} levels in GT1-7 cells.
(a) GT1-7 cells were treated with DMSO or 100 μM IU1 for 48 h. Lysates were analyzed by immunoblotting with anti-PrP (SAF32) and anti-β-actin antibodies. (b) Quantification of PrP^{C} levels in triplicate samples each derived from an independent well performed as in (a). Asterisk indicates significant difference (*P < 0.05). Mean ± SD.

Supplementary Figure S3

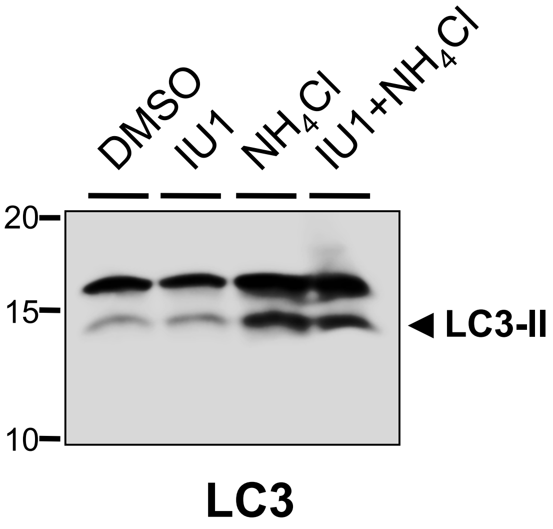

Effect of IU1 treatment on autophagy.
N2a58 cells were treated with DMSO, 100 μM IU1, 10 mM NH_4Cl, or a combination of IU1 and NH_4Cl. Lysates were analyzed by immunoblotting with anti-LC3 antibody.

Supplementary Figure S4

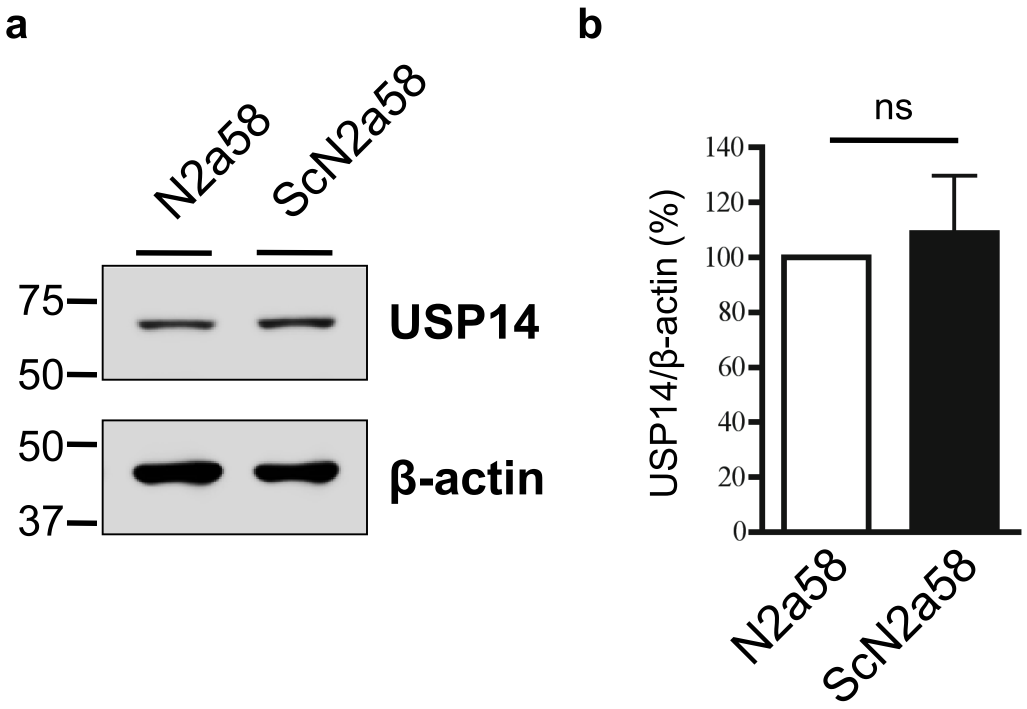

USP14 shows identical protein expression levels in both N2a58 and ScN2a58 cells. (a) N2a58 or ScN2a58 cell lysate were analyzed by immunoblotting with anti-USP14 and anti-β-actin antibodies. (b) Quantification of USP14 protein levels from at least three independent experiments performed as in (a). Mean ± SD. ns: not significant

Supplementary Figure S5

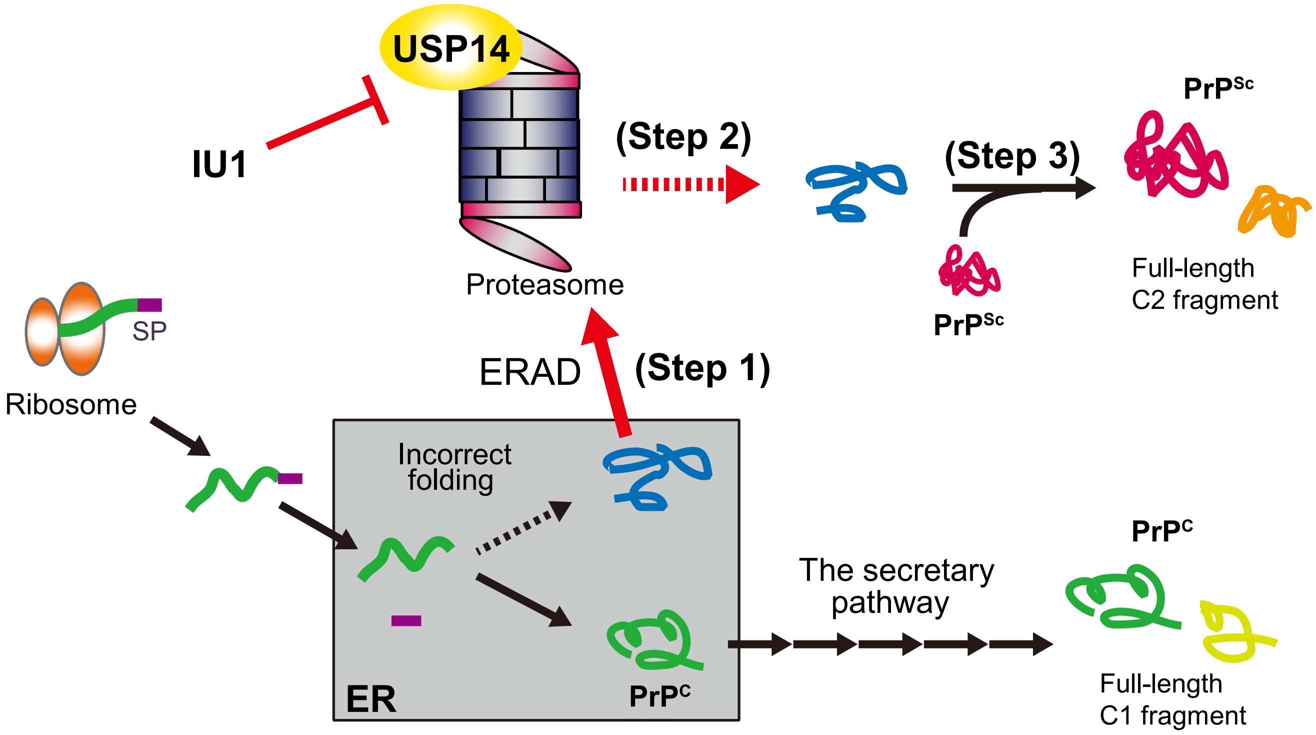

Model for the involvement of USP14 in proteasomal degradation of prion protein. Numbers indicate various steps during USP14 rescues prion protein from proteasomal degradation as described in the discussion. SP: signal peptide, ER: endoplasmic reticulum

Supplementary Figure S6

Figure 1a, PrP^{C}

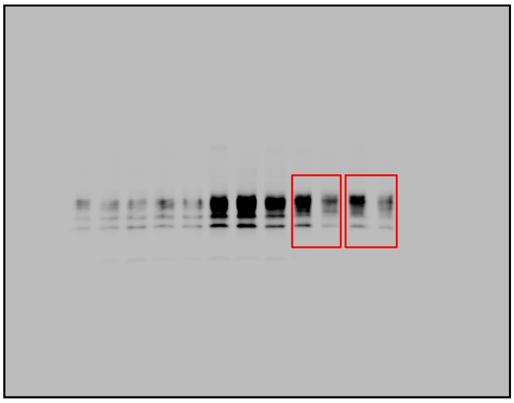

Figure 1f, PrP^{C}

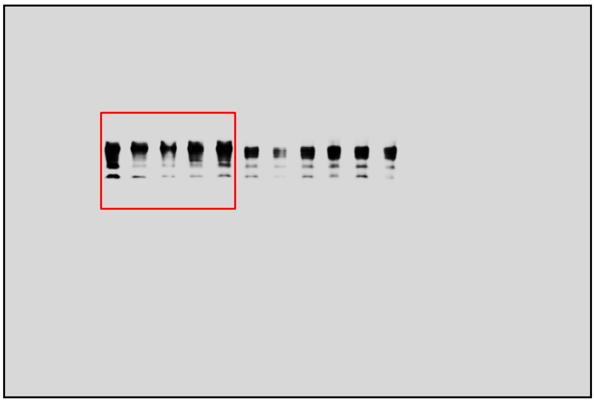

Figure 1a, β-actin

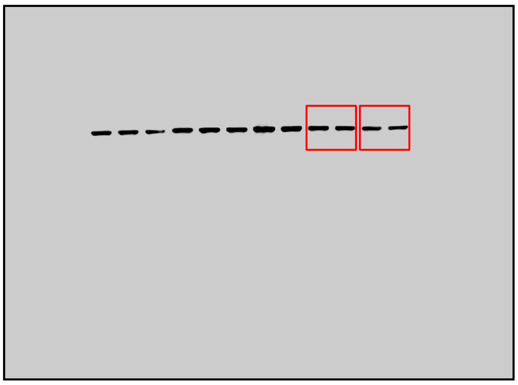

Figure 1f, β-actin

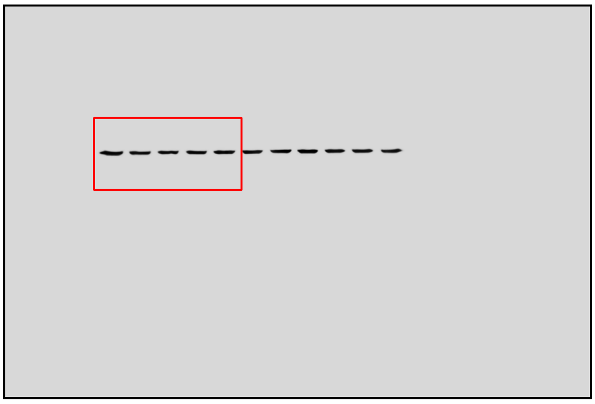

Figure 1c, deglycosylated PrP

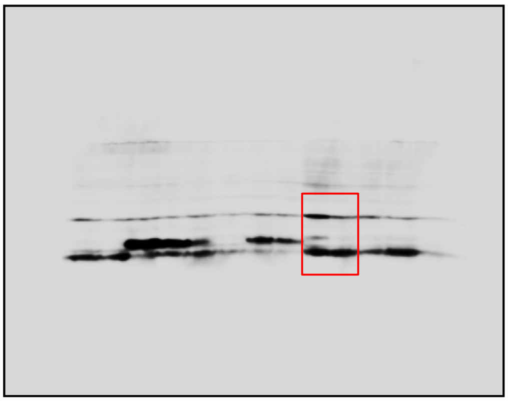

Figure 2a, PrP^{C}

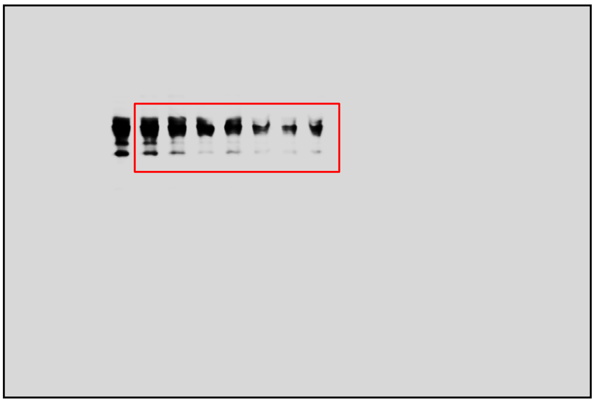

Figure 2a, β-actin

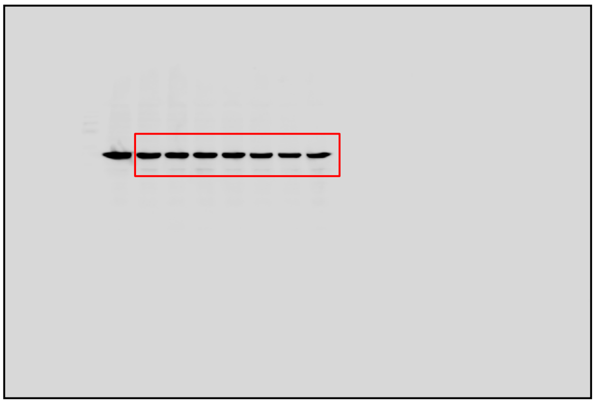

Original uncropped images of immunoblotting of main figures.

Supplementary Figure S6 (continued)

Figure 2b, PrP^C

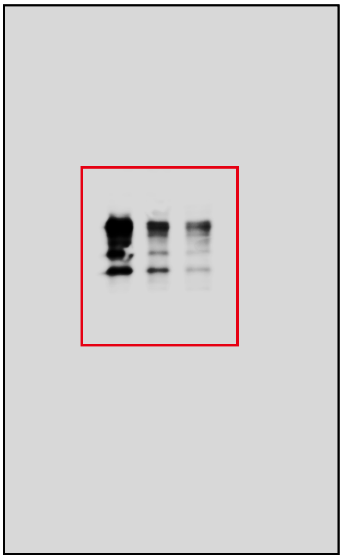

Figure 2b, β-actin

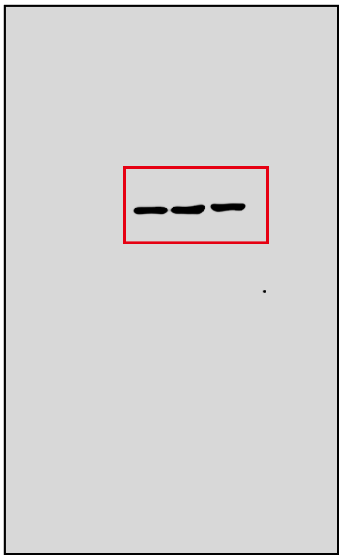

Figure 2d, PrP^C

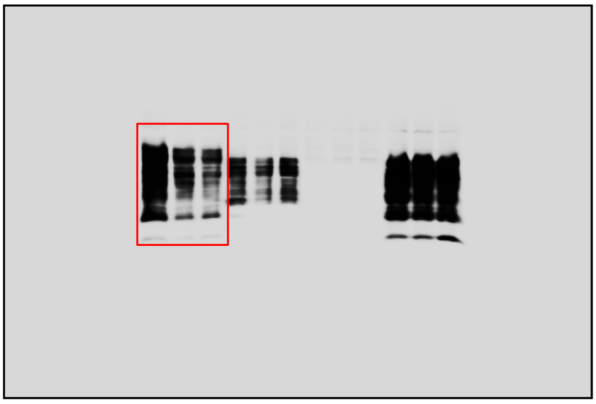

Figure 2d, β-actin

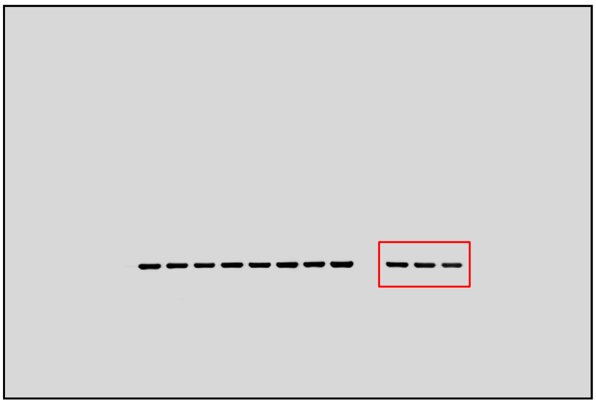

Figure 3a, PK-resistant PrP

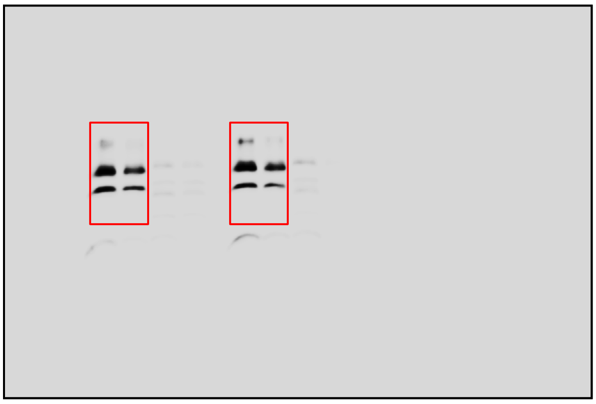

Short exp.

Figure 3c, PK-resistant PrP

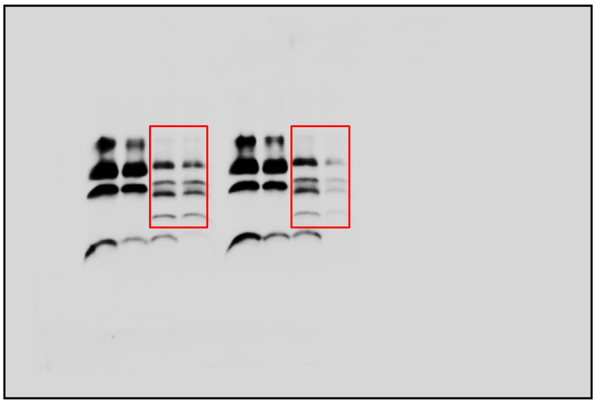

Long exp.

Original uncropped images of immunoblotting of main figures.

Supplementary Figure S6 (continued)

Figure 4a, PrP^{C}

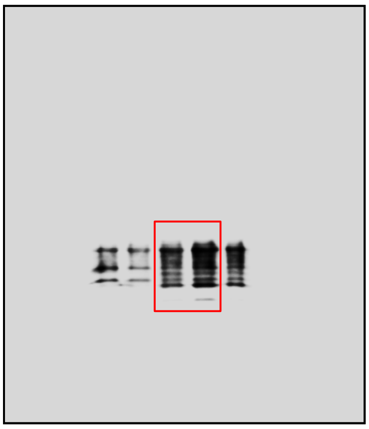

Figure 4c, total PrP

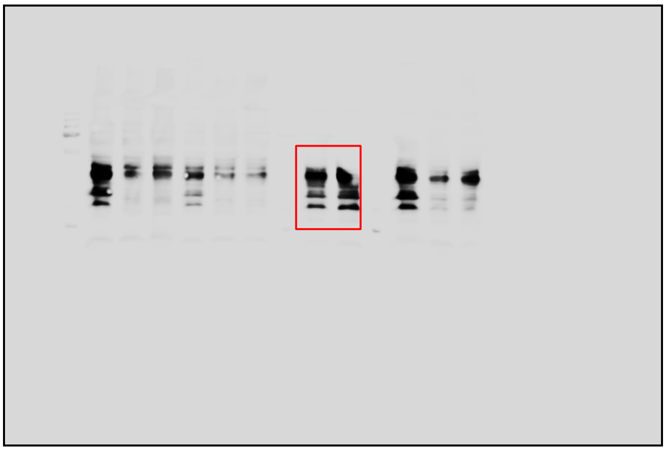

Figure 4a, HA

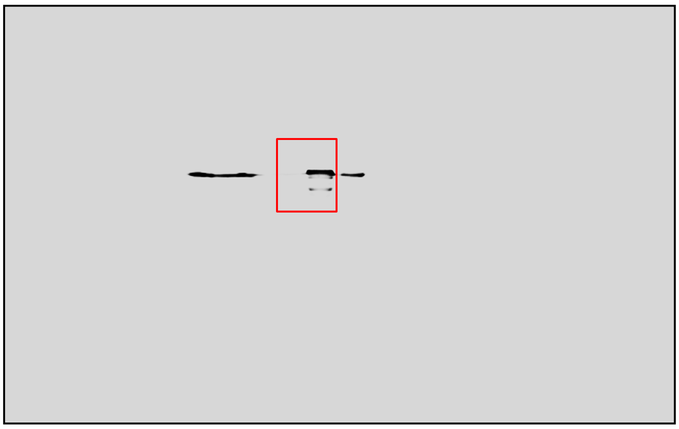

Figure 4c, HA

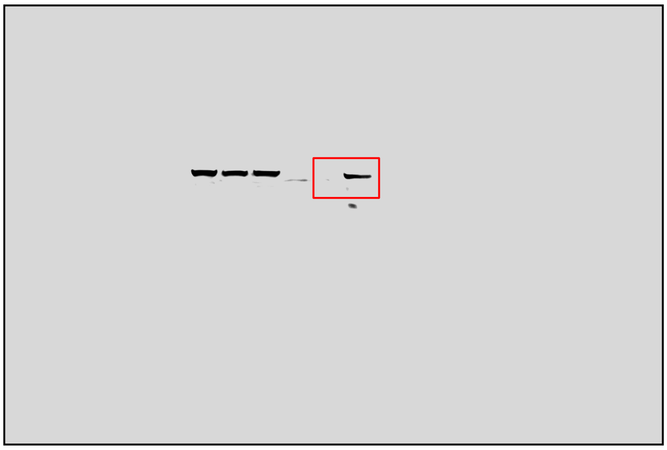

Figure 4a, β-actin

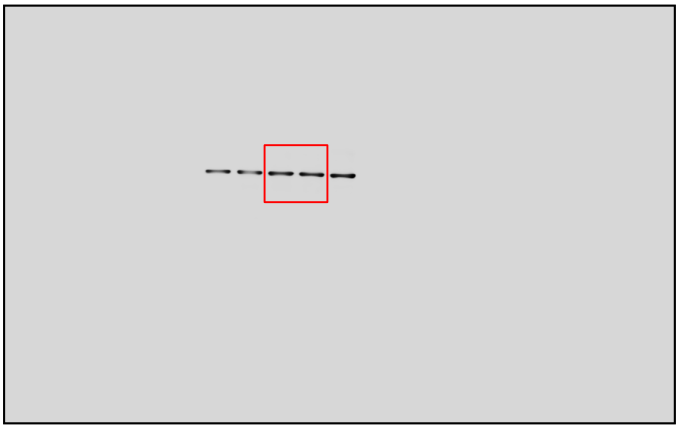

Figure 4c, β-actin

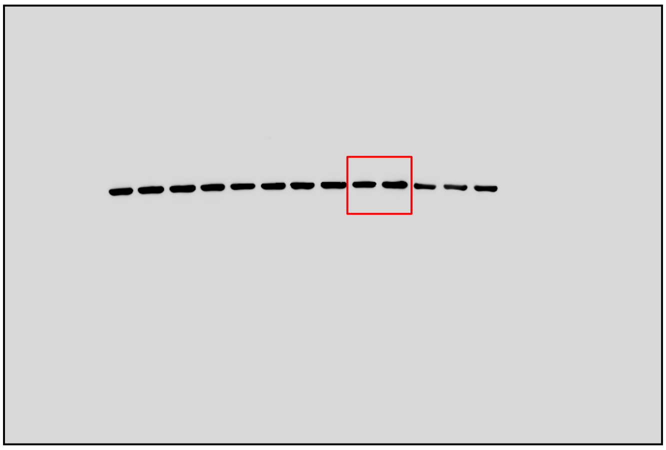

Figure 4b, deglycosylated PrP

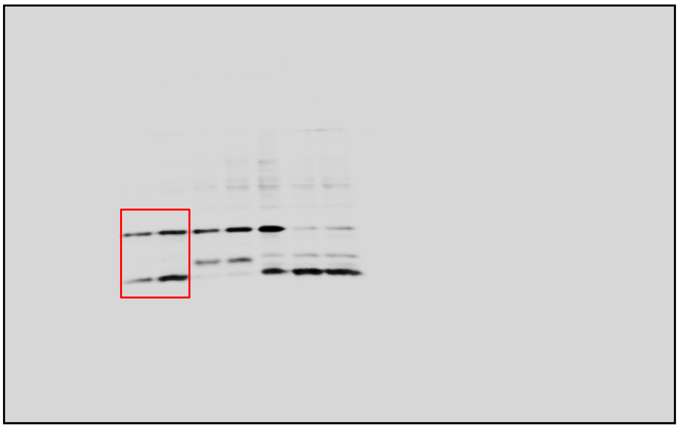

Original uncropped images of immunoblotting of main figures.

Supplementary Figure S6 (continued)

Figure 4d, deglycosylated PrP

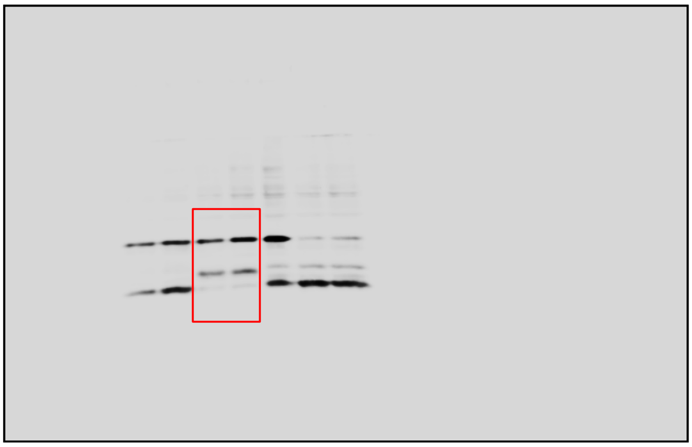

Figure 4e, PK-resistant PrP

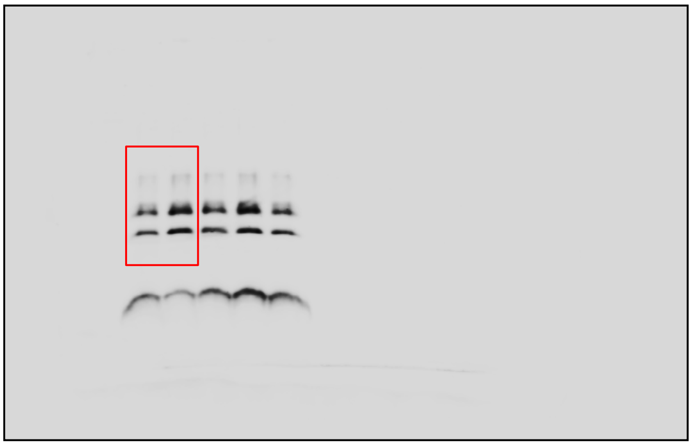

Figure 5b, p53

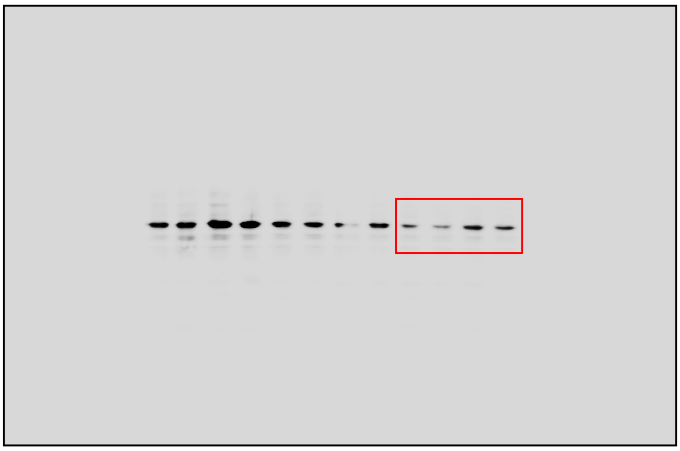

Figure 5c, PK-resistant PrP

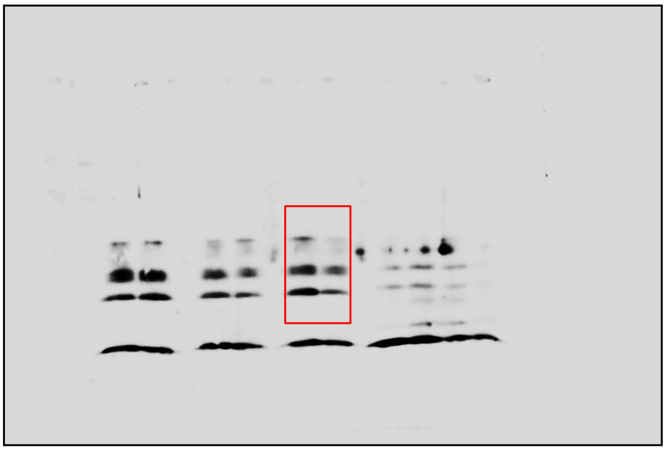

Figure 5c, HA

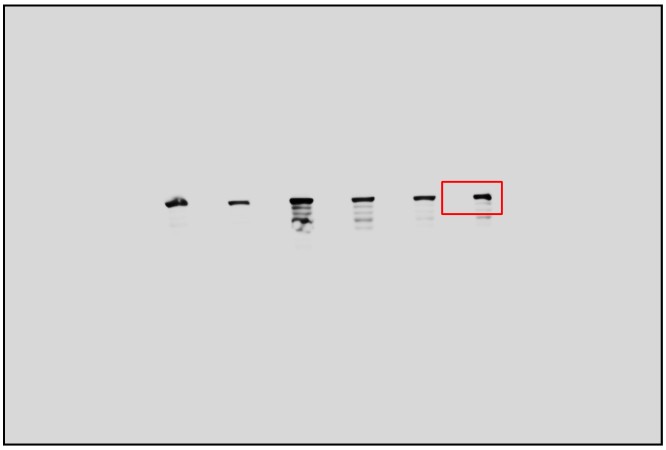

Figure 5c, β-actin

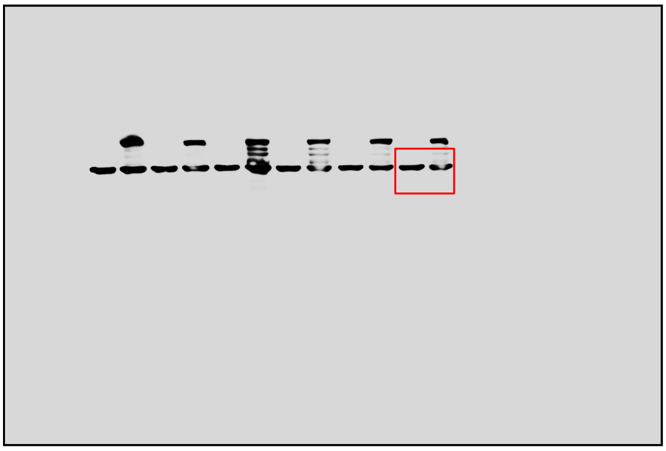

Figure 5e, deglycosylated PrP

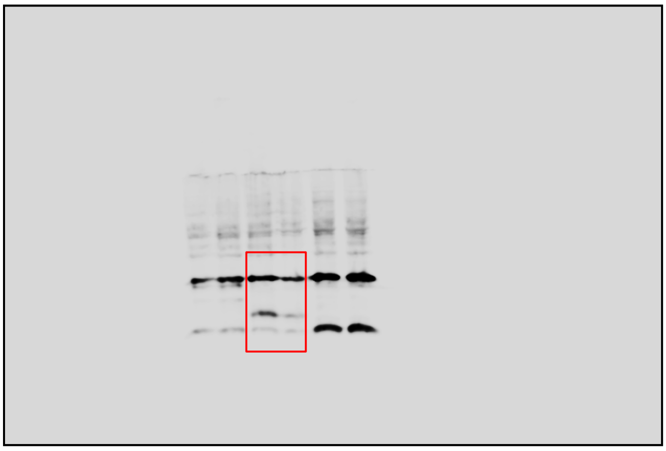

Original uncropped images of immunoblotting of main figures.

Supplementary Figure S6 (continued)

Supplemetary Figure S2a, PrP^{C}

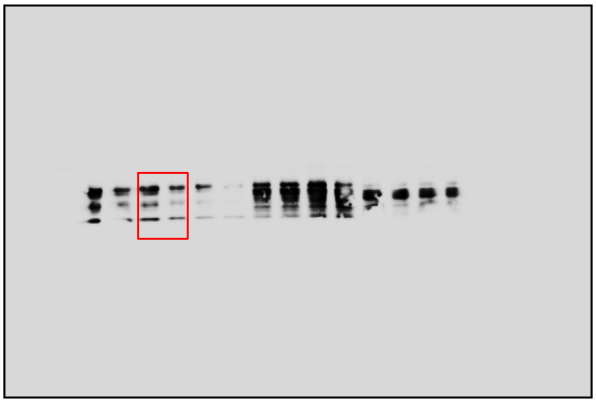

Supplemetary Figure S3, LC3B

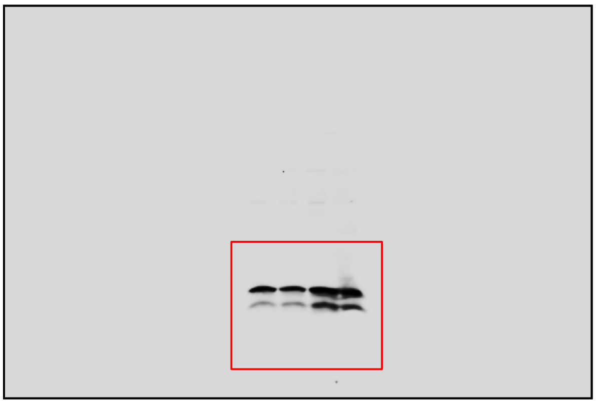

Supplemetary Figure S2a, β-actin

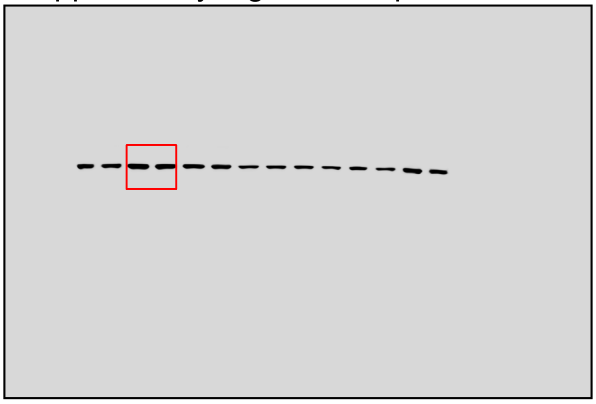

Supplemetary Figure S4a, USP14, β-actin

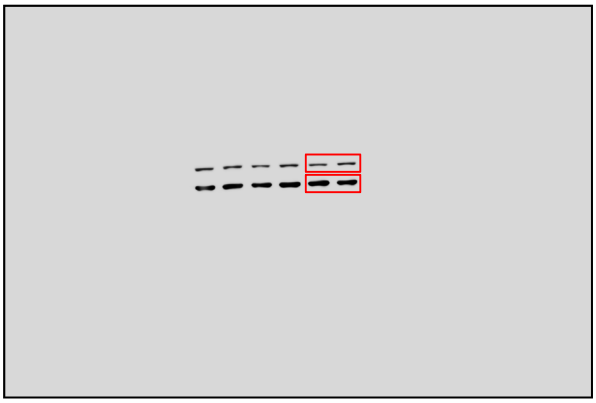

Original uncropped images of immunoblotting of supplemetary figures.
